# A *Ralstonia solanacearum* Effector Targets Splicing Factor *SR34a* to Reprogram Alternative Splicing and Regulate Plant Immunity

**DOI:** 10.3390/plants14040534

**Published:** 2025-02-10

**Authors:** Yunyun Li, Song Kou

**Affiliations:** 1 Hunan Key Laboratory of Plant Functional Genomics and Developmental Regulation, College of Biology, Hunan University, Changsha 410082, China; 2 School of Chemical Engineering and Technology, Xi’an Jiaotong University, Xi’an 710049, China; 19177821895@163.com

**Keywords:** type III secretion system, tomato, RipP2, *SR34a*, acetylation

## Abstract

Alternative splicing is a critical post-transcriptional regulatory mechanism in eukaryotes. While infection with *Ralstonia solanacearum* GMI1000 significantly alters plant alternative splicing patterns, the underlying molecular mechanisms remain unclear. Herein, the effect of the GMI1000 Type III secretion system effectors on alternative splicing in the tomato cultivar Heinz 1706 was investigated. The RNA-seq analysis confirmed genome-wide alternative splicing changes induced by the Type III secretion system in tomato, including 1386 differential alternatively spliced events across 1023 genes, many of which are associated with plant defense. Seven nucleus-localized Type III effectors were transiently expressed in an RLPK splicing reporter system transgenic tobacco, identifying RipP2 as an effector that modulates alternative splicing levels. Sequence analysis, protein–protein interaction assays, and AlphaFold2 structural predictions revealed that RipP2 interacted with the tomato splicing factor *SR34a*. Furthermore, RipP2 acetylated a conserved lysine at position 132 within the SWQDLKD motif of *SR34a*, regulating its splicing pattern in defense-related genes and modulating plant immunity. This study elucidates how the “RipP2-*SR34a* module” influences plant immune responses by regulating the alternative splicing of immune-related genes, providing new insights into pathogen–plant interactions and splicing regulation.

## 1. Introduction

There has been considerable interest in alternative splicing (AS) in recent years, as it is a prevalent and pivotal mechanism of post-transcriptional regulation in biology. AS denotes the process wherein a precursor mRNA (pre-mRNA) undergoes differential splicing events to generate distinct mRNA splice isoforms, culminating in the production of diverse protein variants [1]. This phenomenon substantially augments gene functional diversity and regulatory flexibility, thereby facilitating organismal adaptation to varying environmental cues and physiological demands [2].

In organisms, seven principal types of AS are commonly identified: (1) exon skipping (ES); (2) intron retention (IR); (3) alternative 5′ splice site (A5SS); (4) alternative 3′ splice site (A3SS); (5) alternative promoter usage (AP); (6) alternative polyadenylation (AT); and (7) mutually exclusive exons (MEXs) [3].

The splicing of pre-mRNA is predominantly performed by the spliceosome. The spliceosome is primarily composed of five small nuclear ribonucleoprotein particles (snRNPs), specifically U1, U2, U4/U6, and U5 snRNPs, which are rich in uridine, along with numerous auxiliary proteins. These auxiliary proteins, generally referred to as splicing factors (SFs), are crucial for the regulation of splicing and can be classified into two main categories. The first category includes serine/arginine-rich proteins (SR proteins), which primarily function as splicing activators. The second category comprises heterogeneous nuclear ribonucleoproteins (hnRNPs), which mainly serve as splicing repressors. Together with the snRNPs, these splicing factors constitute the spliceosome and orchestrate the regulation of the splicing reaction [4,5,6,7].

AS regulates various processes in animals and plants. In humans, about 95% of genes undergo AS, mainly via ES, while in plants, around 65% of genes undergo AS, with IR being most common [8,9,10].

In animals, the research on AS primarily focuses on tumorigenesis, neurological disorders, and embryonic development. Studies have shown that AS events in cancer cells are closely associated with malignant transformation, metastasis, and treatment resistance [11,12]. For instance, upon glucose uptake, Tip60 acetylates the splicing factor SRSF5 at K125, thereby promoting the splicing of CCAR1 exons. This generates the CCAR1S isoform, which increases glucose consumption and acetyl-CoA production, thereby promoting tumor growth [13]. In Parkinson’s disease, genes such as PARK2 and SNCAIP undergo abnormal splicing [14,15]. Moreover, during development, the regulation of AS events enables the generation of diverse cell and tissue types, facilitating organ development and tissue differentiation [16,17,18]. These studies provide critical insights into the functions and regulatory mechanisms of AS.

Over the past few years, substantial advancements have been made in understanding AS in plants. AS regulation is integral to plant development, stress responses, and disease resistance, facilitating plant adaptation to diverse environmental condition, especially in the immune process of plants against pathogens [19,20,21,22,23].

Early studies have shown that in plant resistance responses to pathogens, the precise and rapid regulation of AS mechanisms can adjust the ratio of functional and non-functional transcripts of defense-related genes, including pattern recognition receptor (PRR) genes, resistance (R) genes, and specific splicing factors (SFs), which play a crucial role in enabling plants to mount a robust immune response [24,25,26,27,28,29,30,31].

Notably, the most recent work has revealed that in the prolonged battle between plants and pathogens, certain pathogens have evolved specific effectors that reprogram host pre-mRNA splicing to disrupt plant immunity and facilitate pathogen infection. Examples include the *Pseudomonas syringae* type III-secreted effector HopU1, which targets several *Arabidopsis* RNA-binding proteins, such as GRP7, resulting in reduced FLS2 protein levels and suppression of host immunity [32]. The avirulent effector PsAvr3c from *Phytophthora sojae* interacts with soybean GmSKRPs, influencing the splicing of host pre-mRNA and facilitating disease progression [33]. Similarly, the RXLR effector SRE3 from *Phytophthora infestans* interacts with the tomato splicing factor U1-70K, altering the AS of both susceptibility and resistance genes to promote infection [34]. *Phytophthora* effector PSR1 binds to PINP1, impairing its activity, which leads to widespread PINP1-mediated AS events and inhibition of sRNA production, ultimately compromising plant immunity [35]. In another case, wheat streak rust (*Pst*) secretes the effector *Pst*-A23, which binds to RNA motifs at the splicing sites of wheat genes *TaXa21-H* and *TaWRKY53*, disrupting AS and weakening the plant’s defense [36]. Additionally, the NIa-Pro, encoded by SCMV, disrupts the splicing activity of maize ZmU2AF65B., thereby impairing mRNA surveillance and promoting viral infection [37].

Despite AS being crucial for plant immunity, the molecular mechanisms by which pathogen effectors disrupt host AS to compromise immunity remain incompletely unclear.

*Ralstonia solanacearum* is a soil-dwelling bacterium that infects over 450 plant species from more than 50 plant families, resulting in vascular wilt and plant death [38,39].

The model strain GMI1000 threatens major crops like peppers, tomatoes, and tobacco by invading the roots. Its pathogenicity is driven by cell wall-degrading enzymes, extracellular polysaccharides, effectors, and bacterial motility, disrupting vascular function and causing wilting [40,41]. Its Type III secretion system (T3SS) is a key virulence factor, enabling the injection of multiple effectors into host cells to suppress immunity and disrupt cellular functions. Various T3SS effectors in *R. solanacearum* employ diverse strategies to infect and suppress host immunity [41,42].

While previous studies have shown that infection by the *R. solanacearum* strain GMI1000 significantly alters AS levels in the non-native host *Arabidopsis thaliana* [43], the precise underlying mechanisms driving these changes are yet to be fully characterized.

Therefore, this study explored how GMI1000 T3SS effectors regulate AS in the tomato reference cultivar Heinz 1706.

Herein, the splicing regulatory function of T3SS of GMI1000 was initially validated using RNA-seq and RT-qPCR analysis, particularly focusing on its regulation of defense-related genes in the spliceosome pathway.

Subsequently, by transiently expressing seven subcellular nucleus-localized T3SS effectors in *Nicotiana benthamiana* plants that had been engineered to express the splicing reporter *RLPK-LUC*, the effector RipP2, a previously reported acetyltransferase, was identified as capable of inducing changes in AS levels.

Furthermore, through sequence analysis, AlphaFold2 structural predictions, and a series of biochemical and molecular experiments, the interaction between RipP2 and the tomato splicing factor *SR34a* was revealed. Finally, RipP2 was found to acetylate the conserved lysine residue at position 132 (K132) within the SWQDLKD motif of *SR34a*, affecting the splicing pattern of *SR34a* on defense-related genes and regulating plant immunity.

These findings not only contribute to the understanding of how plants respond to pathogen infection through AS, but also reveal new strategies by which pathogens utilize their effector proteins to interfere with plant signaling and gene expression regulatory mechanisms.

## 2. Results

### 2.1. The T3SS of R. solanacearum GMI1000 Contributes to Its Pathogenicity and Regulates the Alternative Splicing of Defense-Related Genes in Tomato

Previous studies have demonstrated that numerous plant pathogens have evolved effectors to regulate AS in plants [32,33,34,35,36,37]. Additionally, the T3SS of *R. solanacearum*, as a key virulence factor, is capable of secreting a variety of proteins to attack the host [19,20,21,22,23].

To analyze the role of T3SS in the virulence of the model strain GMI1000 and its potential regulatory effects on host AS, two T3SS mutants of strain GMI1000 were used: the transcriptional regulation mutant GMI1000 Δ*hrpB* and the membrane channel mutant GMI1000 Δ*hrcV* (hereinafter referred to as Δ*hrpB* and Δ*hrcV*, respectively). These mutants, along with the wild-type strain, were inoculated onto the natural host tomato Heinz1706 and experimental host *A. thaliana* ecotype Col-0 to assess their pathogenicity.

**Figure 1:**
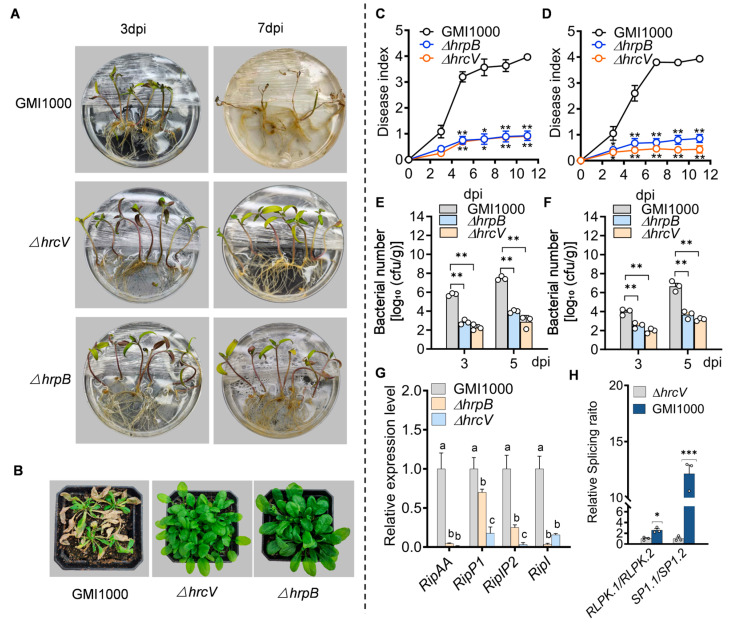
Effects of *R. solanacearum* strain GMI1000 and its T3SS mutants (Δ*hrcV* and Δ*hrpB*) on host plant pathogenicity and alternative splicing. (**A**) Disease symptoms in tomato seedlings infected with GMI1000, Δ*hrcV*, and Δ*hrpB* at 3 and 7 days post-inoculation (dpi), showing significantly reduced pathogenicity in the mutants. (**B**) Disease symptoms in *A. thaliana* Col-0 infected with GMI1000 and the mutants, with mutant-infected plants displaying markedly reduced symptoms. (**C**,**D**) Disease index over time in tomato (**C**) and *A. thaliana* Col-0 (**D**) infected with GMI1000, Δ*hrcV*, and Δ*hrpB*. GMI1000-infected plants show a rapid increase in disease index, while mutant-infected plants maintain a consistently low index. Statistical analysis in (**C**–**F**) was conducted using a two-sample *t*-test; * *p* < 0.05 and ** *p* < 0.01. (**E**,**F**) Bacterial load in the roots of tomato (**E**) and *A. thaliana* (**F**) at 3 and 5 dpi, with significantly higher levels in GMI1000-infected plants compared to the mutants. (**G**) Relative expression levels of type III effectors RipAA, RipP1, RipP2, and RipI in tomato roots, normalized to GMI1000 (set to 1). Different letters indicate statistically significant differences (*p* < 0.05; one-way ANOVA followed by Duncan’s multiple range test). (**H**) Relative splicing ratio of defense-related genes (*RLPK*1/*RLPK*.2 and *SP1*.1/*SP1*.2) in tomato roots infected with GMI1000 and Δ*hrcV*. Statistical significance was assessed using a two-tailed *t*-test: *** *p* < 0.001, ** *p* < 0.01, and * *p* < 0.05. Error bars represent the average ± standard deviation (SD). All experiments were performed in triplicate, yielding consistent results.

As shown in Figure 1A, tomato seedlings infected with wild-type *R. solanacearum* GMI1000 begin to show obvious disease symptoms 3 days after inoculation (3 dpi), including obvious yellowing and wilting, and completely wilted 7 days after inoculation. However, tomato seedlings infected with Δ*hrcV* and Δ*hrpB* mutants had no obvious disease symptoms. Similarly, *A. thaliana* plants displayed marked disease symptoms upon GMI1000 infection, while Δ*hrcV* and Δ*hrpB* infections resulted in no observable symptoms (Figure 1B). The disease index of tomato (Figure 1C) and *Arabidopsis* (Figure 1D) plants was measured at different time points (2 dpi–11 dpi). The results show that the disease index of plants infected with GMI1000 increases rapidly in the early stage of infection, whereas those infected with Δ*hrcV* or Δ*hrpB* mutants have significantly lower disease indices.

Bacterial load assays at 3 and 5 dpi revealed that GMI1000 exhibited significantly higher colonization in tomato (Figure 1E) and *A. thaliana* roots (Figure 1F) compared to Δ*hrcV* and Δ*hrpB*, indicating that T3SS is crucial for effective root colonization in both hosts. These results demonstrate that T3SS is essential for pathogenicity and successful colonization by *R. solanacearum*.

Furthermore, the expression levels of key T3SS effectors RipAA, RipP1, RipP2, and RipI in tomato cultivar Heinz 1706 were assessed using quantitative real-time PCR (RT-qPCR). Compared to GMI1000, the Δ*hrpB* mutant showed partially reduced effector expression, while Δ*hrcV* nearly abolished it (Figure 1G). Therefore, to ensure a complete assessment of T3SS inactivation, Δ*hrcV* was selected for subsequent experiments to investigate the role of T3SS in regulating AS.

To preliminarily explore whether GMI1000 regulates host defense-related AS via T3SS, two defense-related tomato genes with intron retention (IR) events were selected, receptor-like protein kinase (*RLPK*) and E3 ubiquitin ligase (*SP1*), for AS validation. *RLPK* and *SP1* play critical roles in plant immunity, with *RLPK* involved in pathogen recognition and signaling, and *SP1* in protein processing and signaling. The *RLPK* and *SP1* genes undergo significant AS changes during *Phytophthora* infection [34], which affect their functions in immune response. Their moderate expression levels made them suitable for RT-qPCR to quantify AS isoform ratios, where *RLPK.1/RLPK.2* and *SP1*.1/*SP1*.2 represent the ratio of intron-splicing isoforms to intron-retaining isoforms. The results of RT-qPCR show that, under GMI1000 infection, the splicing ratios of *RLPK.1/RLPK.2* and *SP1*.1/*SP1*.2 significantly increase, with *RLPK.1/RLPK.2* approximately three-times higher and *SP1*.1/*SP1*.2 about twelve-times higher than in Δ*hrcV*-infected samples. These findings suggest that T3SS may regulate the splicing pattern of *RLPK* and *SP1*, favoring the production of functional, intron-free isoforms, thereby modulating host immune responses.

Collectively, these findings indicate that T3SS is crucial for *R. solanacearum* GMI1000 pathogenicity, influencing both bacterial colonization and virulence. Furthermore, it likely modulates the AS of specific defense-related genes.

### 2.2. T3SS of R. solanacearum GMI1000 Results in Genome-Wide AS Changes in Tomato

As a natural host for *R. solanacearum*, tomato provides an optimal model for studying the regulatory impact of the T3SS on host defense gene AS, as indicated by the preliminary experimental findings.

To further elucidate the regulatory role of the T3SS in the AS of tomato genes, RNA sequencing was performed on Heinz 1706 tomato plants inoculated with either GMI1000 or the T3SS mutant Δ*hrcV*, with each treatment replicated three times. Differentially expressed genes (DEGs) and differentially alternatively spliced genes (DASGs) in tomato following pathogen infection were analyzed. A total of 257,709,242 quality-controlled reads were obtained through RNA-seq (Figure S1), which were aligned to the tomato reference genome and assembled into transcript sequences as detailed in the methods.

**Figure 2:**
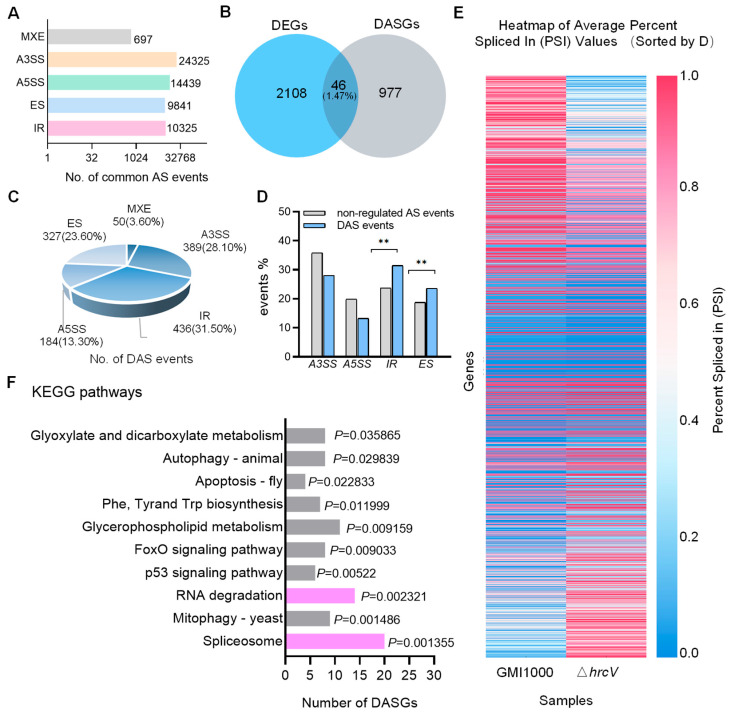
Regulation of AS in tomato by T3SS. (**A**) The distribution of four major types of alternative splicing (AS) events was analyzed under GMI1000 and Δ*hrcV* infection conditions. (**B**) Venn diagram illustrating the overlap between DEGs and DASGs, showing a limited overlap (1.47%), which suggests a relative independence between transcriptional regulation and splicing regulation. (**C**) Proportional distribution of different types of DAS events, with IR (31.5%) and A3SS (28.1%) being the most prevalent. (**D**) Comparison between DAS and non-regulated AS events, showing a significant increase in IR and ES events among DAS events (one-tailed *t*-test, ** *p* < 0.01). (**E**) Heatmap representing the Percent Spliced In (PSI) values, displaying the PSI distribution for 1386 DAS events under Δ*hrcV* and GMI1000 conditions. The heatmap displays genes as rows and samples as columns, with color gradients corresponding to the PSI values. (**F**) Top 10 significantly enriched KEGG pathways among DASGs, including the spliceosome, mitophagy, and RNA degradation pathways (enrichment analysis, *p* < 0.05).

According to the tomato reference genome annotations, common AS events in GMI1000- and *ΔhrcV*-infected samples were identified. Among the commonly identified AS events across the two samples, different types of AS events exhibited distinct proportional distributions. The five major types of AS events were as follows: A3SS accounted for 40.80% (24,325 events), A5SS accounted for 24.22% (14,439 events), IR accounted for 17.32% (10,325 events), ES accounted for 16.50% (9,841 events), and MXE accounted for 1.17% (697 events) (Figure 2A) (Table S1).

Differential AS analysis was performed using rMATS/rMATS.4.1.1 to identify different AS events, with the options described in the Methods Section. AS events with *p* values < 0.05 were classified as differentially alternatively spliced (DAS) events [44]. At the same time, by adding the –novel SS parameter, rMATS can detect splicing sites that are not recorded in the reference genome annotation file, which helps to identify more potential AS events.

Comparing GMI1000 to Δ*hrcV*, 2154 DEGs and 1023 DASGs were detected. Analysis revealed a low overlap (1.47%) between DEGs and DASGs, with only 46 genes overlapping (Figure 2B, Table S2). This low overlap suggests distinct regulatory mechanisms between gene expression and AS, whereby transcriptional regulation is primarily controlled by transcription factors and regulators, while AS is driven by spliceosome composition and function. Therefore, transcriptional and splicing regulation may play unique roles in cellular responses to external stimuli and internal signals.

A total of 1023 DASGs were identified, encompassing 1386 AS events, including 389 A3SS, 184 A5SS, 327 ES, 436 IR, and 50 MXE events (Figure 2C, Table S2).

Further comparative analysis of AS events between differential and non-differential conditions focused on the four primary splicing types: IR, ES, A3SS, and A5SS. The largest AS change in GMI1000-infected samples relative to Δ*hrcV* was in IR events (+32.63%), followed by ES (+25.70%). Conversely, A3SS and A5SS exhibited relative decreases of −21.59% and −33.20%, respectively (Figure 2D, Table S3). This indicated that GMI1000 T3SS preferentially modulated IR and ES events, particularly IR, warranting further investigation into the biological significance of IR.

To obtain a comprehensive view of AS changes, the analysis of splicing events was performed using Percent Spliced In (PSI) values, with a threshold of |ΔPSI| > 0.05 to quantify relative changes (Figure 2E, Table S4). PSI represents the proportion of transcripts in which a specific AS event, including exon inclusion or intron retention, is present relative to all transcripts involving that splicing event. PSI heatmaps of 1386 DAS events under GMI1000 and Δ*hrcV* conditions revealed substantial splicing differences, with lower PSI values in GMI1000 and higher values in Δ*hrcV*, indicating that T3SS significantly impacts AS patterns in tomato.

In conclusion, these findings demonstrate that the T3SS of *R. solanacearum* GMI1000 markedly induces AS changes in tomato, particularly through increased Differential IR and ES events.

### 2.3. Genes Undergoing AS Changes Are Enriched in the Spliceosome and RNA Degradation Pathways

To further investigate the functions of tomato genes associated with common types of DAS events identified in both samples, KEGG pathway enrichment analysis was conducted. The KEGG analysis revealed that the DASGs were significantly enriched in pathways, including the “spliceosome”, “glycerophospholipid metabolism”, “RNA degradation”, and “glycosaminoglycan degradation” (Figure 2F, Table S5). Among these, the spliceosome and RNA degradation pathways were particularly prominent.

The spliceosome pathway, which is closely associated with pre-mRNA splicing, showed significant enrichment (*p* = 0.001355), suggesting that the T3SS of GMI1000 may affect the host’s mRNA splicing mechanism by modulating spliceosome component activity.

Additionally, significant enrichment in the RNA degradation pathway (*p* = 0.002321) implies that T3SS may regulate genes involved in RNA degradation, potentially influencing the degradation rate and stability of host pre-mRNAs to more precisely control transcription and optimize immune responses. The enrichment of these two pathways indicates that the pathogen may synergistically impact host defense mechanisms by regulating AS and mRNA stability. Such multi-layered regulatory mechanisms may provide adaptive advantages in plant–pathogen interactions.

Notably, the majority of genes in the spliceosome and RNA degradation pathways contained IR events, with many of these genes being defense related. Additionally, pathways related to autophagy, including “mitophagy (Mitophagy—yeast)” and “autophagy (Autophagy—animal)”, were significantly enriched, suggesting that T3SS may also influence host defense mechanisms against cellular pathogen invasion by modulating the autophagy pathway.

To validate the DAS events identified in the RNA-seq data, nine functionally characterized genes were selected from the pathways significantly enriched in the KEGG analysis, including eight with IR events and one with an ES event (Table S2), and their isoform transcript levels were quantified using RT-qPCR.

**Figure 3:**
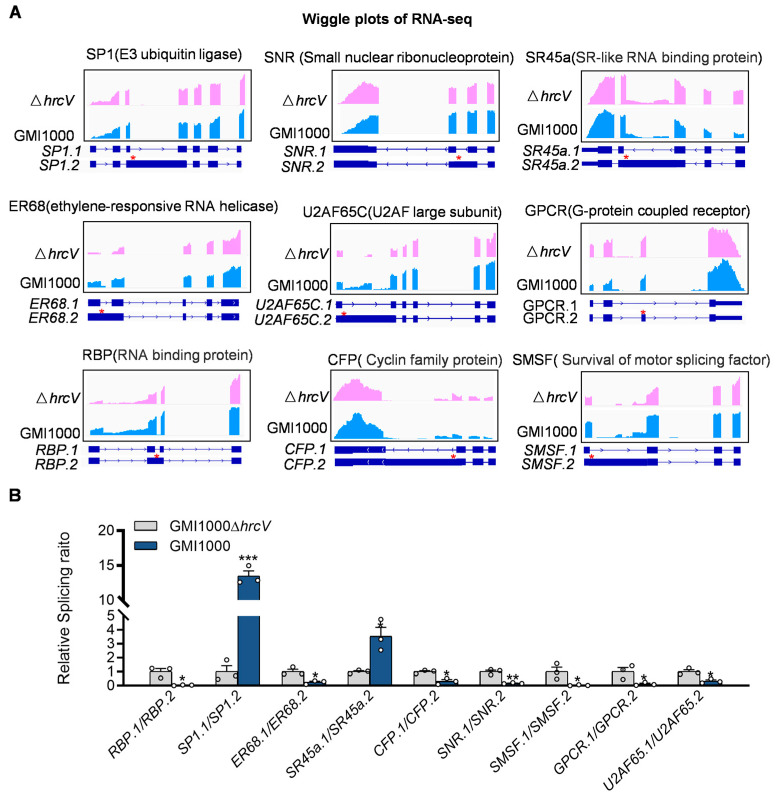
Validation of AS events in selected genes based on RNA-seq data under GMI1000 and Δ*hrcV* infection conditions. (**A**) RNA-seq data wiggle plots for nine functionally characterized genes, including eight IR and one exon skipping ES events. Genes include *SP1* (E3 ubiquitin ligase), *SNR* (small nuclear ribonucleoprotein), *SR45a* (SR-like RNA binding protein), *ER68* (ethylene-responsive RNA helicase), *U2AF65C* (U2AF large subunit), *GPCR* (G-protein coupled receptor), *RBP* (RNA binding protein), *CFP* (cyclin family protein), and *SMSF* (survival of motor splicing factor). Isoforms under Δ*hrcV* (pink) and GMI1000 (blue) conditions are shown, with splicing events annotated below. Gene structure diagrams are displayed at the bottom of each wiggle plot, representing different splice isoforms, with asterisks indicating regions of intron retention or exon skipping. (**B**) Relative splicing ratios for selected IR and ES events determined by RT-qPCR. Statistical significance was assessed using a two-tailed *t*-test: *** *p* < 0.001, ** *p* < 0.01, and * *p* < 0.05. Error bars represent the average ± standard deviation (SD).

These nine DAS events involved genes encoding E3 ubiquitin ligases, G protein-coupled receptors, cyclins, and proteins related to RNA splicing and RNA degradation. Figure 3A illustrates the expression patterns of these genes, including *SP1* (E3 ubiquitin ligase, *Solyc06g084360.2*), *SNR* (small nuclear ribonucleoprotein, *Solyc02g062240.2*), *SR45a* (SR-like RNA binding protein, *Solyc02g061840.2*), *ER68* (ethylene-responsive *RNA* helicase, *Solyc12g044860.1*), *U2AF65C* (U2AF large subunit, *Solyc02g085570*.2), *GPCR* (G protein-coupled receptor, *Solyc08g061260.2*), *RBP* (RNA-binding protein, *Solyc04g014460.2*), *CFP* (cyclin family protein, *Solyc07g032480.2*), and *SMSF* (motor neuron splicing factor, *Solyc01g103020.2*) (Figure 3A).

RT-qPCR data indicate significant alterations in the AS patterns of these genes. Regarding IR events, the IR events in GMI1000-infected samples were significantly altered compared to Δ*hrcV*. Specifically, the splicing ratios of intron-spliced to intron-retained isoforms of *SP1* and *SR45a* increased significantly, while those of *SP1*, *SNR*, *U2AF65C*, *SMSF*, *CFP*, and *ER68* decreased significantly (Figure 3B). For the ES event, significant differences were observed in the transcript levels of *GPCR* isoforms, including exon-included and exon-skipped variants’ isoforms (Figure 3B). The results of the RT-qPCR are in concordance with the RNA-seq data.

Together, these findings indicate that the T3SS of GMI1000 can modulate IR events in numerous tomato genes associated with RNA splicing, defense, and other critical regulatory processes

### 2.4. Genes with Altered Intron Retention Levels in the Spliceosome Pathway Are Associated with Defense Responses

IR typically introduces premature stop codons, resulting in aberrant mRNA transcripts that either produce truncated proteins or are degraded via nonsense-mediated RNA decay (NMD) in the cytoplasm or targeted for degradation by nuclear RNA surveillance mechanisms [45].

To gain deeper insights into the functions of alternative protein isoforms generated by IR changes in key regulatory genes, four genes involved in the spliceosome and RNA degradation pathways were selected, with known functions and variations in splicing ratios, for functional analysis: *RBP* (RNA-binding protein), *SNR* (small nuclear ribonucleoprotein), *ER68* (ethylene-responsive RNA helicase), and *U2AF65C* (U2AF large subunit) (Figure 3A,B).

In *N. benthamiana*, eight GFP-tagged splice isoforms of these four genes (Table S6) were transiently expressed and subsequently inoculated the plants with the pathogenic *R. solanacearum* strain CQPS-1, which can infect *N. benthamiana*. Among the isoforms, transcripts of *RBP.1*, *ER68.1*, *SNR.1*, and *U2AF65C.1* produced functional proteins, whereas transcripts of *RBP.2*, *ER68.2*, *SNR.2*, and *U2AF65C.2* generated truncated, non-functional proteins due to intron retention or were directed toward RNA degradation pathways. The expression of these protein isoforms was confirmed through immunoblotting (Figure S2).

**Figure 4:**
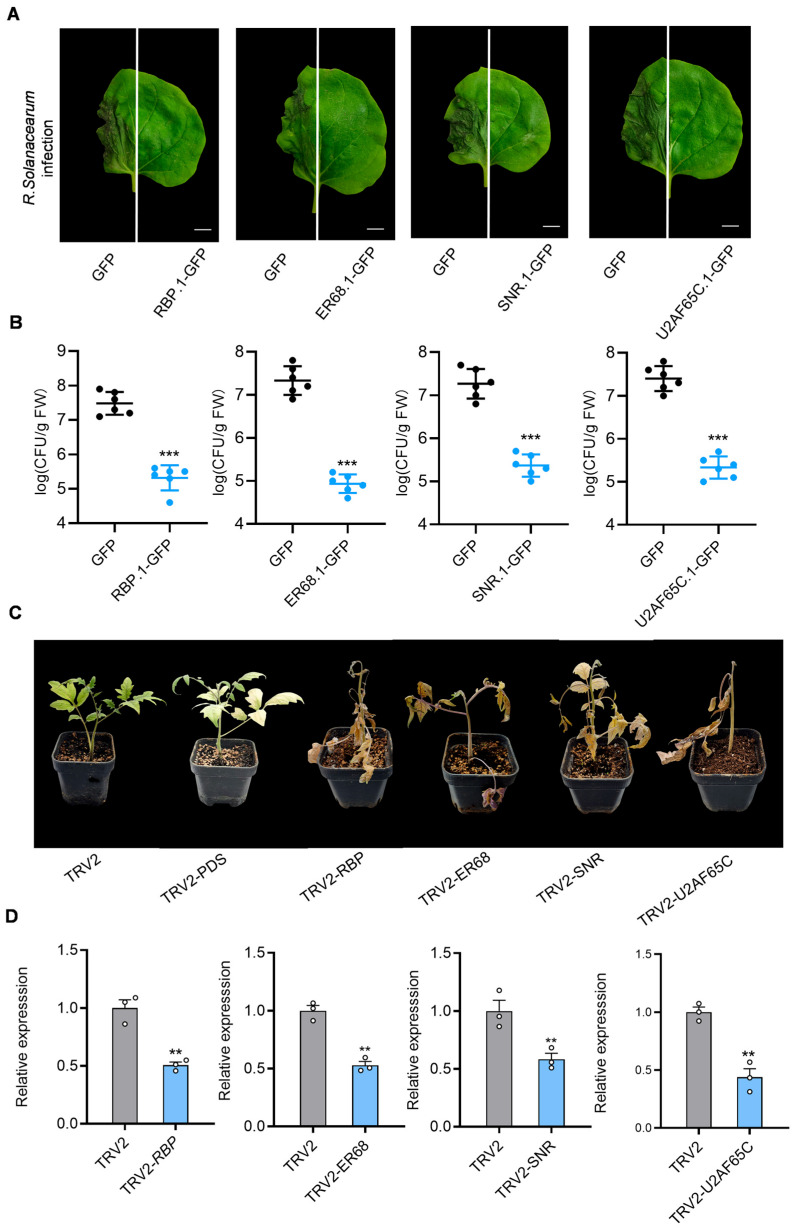
Functional role of spliceosome-related genes with intron retention (IR) in defense against *R. solanacearum*. (**A**) Transient expression of GFP-tagged splice isoforms of RBP, ER68, SNR, and U2AF65C in *N. benthamiana* leaves, which were subsequently inoculated with *R. solanacearum* CQPS-1. Leaves expressing *RBP*.1, *ER68*.1, *SNR*.1, and *U2AF65C.1* exhibited reduced disease symptoms compared to the GFP control, suggesting enhanced resistance. (**B**) Colony-forming unit (CFU) counts in *N. benthamiana* leaves show significantly lower bacterial loads in *RBP.1*, *ER68.1*, *SNR.1*, and *U2AF65C.1* overexpression groups compared to the GFP control (*p* < 0.05), indicating restricted pathogen colonization. (**C**) Gene silencing of RBP, ER68, SNR, and U2AF65C in tomato plants was performed using virus-induced gene silencing (VIGS). Silenced plants (TRV2-*RBP*, TRV2-*ER68*, TRV2-*SNR*, and TRV2-*U2AF65C*) displayed increased disease symptoms post-inoculation with *R. solanacearum*, highlighting reduced resistance. (**D**) RT-qPCR confirmation of gene silencing efficiency, showing approximately a 50% reduction in target gene expression in VIGS-treated groups relative to the TRV2 control (*p* < 0.01). Statistical significance was assessed using a two-tailed *t*-test: *** *p* < 0.001, ** p < 0.01. Error bars represent the average ± standard deviation (SD). All experiments were performed in triplicate, yielding consistent results.

Upon *R. solanacearum* inoculation, the areas overexpressing *RBP.1*, *ER68.1*, *SNR.1*, and *U2AF65C.1* in *N. benthamiana* leaves showed inhibited *R. solanacearum* CQPS-1 infection compared to the GFP control, while the overexpression of *RBP.2*, *ER68.2*, *SNR.2*, and *U2AF65C.2* had no significant effect (Figure 4A and Figure S2).

To evaluate disease resistance, colony-forming units (CFUs) of *R. solanacearum* were measured in infected leaves. The results show that CFU counts are significantly lower in the overexpression groups of *RBP.1*, *ER68.1*, *SNR.1*, and *U2AF65C.1* compared to the GFP control group, suggesting that overexpressing these functional isoforms may limit pathogen colonization and enhance resistance to infection (Figure 4B).

To further verify the roles of these genes in *R. solanacearum* infection, virus-induced gene silencing (VIGS) was utilized in tomato for silencing RBP, ER68, SNR, and U2AF65C, and we subsequently performed infection experiments with *R. solanacearum*. The expression of these genes in the TRV2.*RBP*, TRV2.*ER68*, TRV2.*SNR*, and TRV2.*U2AF65C* groups, as measured by RT-qPCR, was approximately 50% of that in the TRV2 control group, indicating effective gene silencing by VIGS.

Compared to the TRV2 empty vector control and positive control TRV2-*PDS*, tomato plants in the TRV2-*RBP*, TRV2-*ER68*, TRV2-*SNR*, and TRV2-*U2AF65C* groups exhibited earlier and more severe disease symptoms, including leaf wilting and chlorosis, seven days after inoculation with *R. solanacearum* GMI1000. This suggests that silencing these genes compromised tomato resistance to *R. solanacearum*.

These findings imply that the four genes involved in spliceosome pathway likely function as positive regulators of immunity against *R. solanacearum*, playing critical roles in enhancing host resistance to infection. During GMI1000 infection, the T3SS may interferes with the normal splicing of these immune-related positive regulators within the spliceosome pathway, significantly reducing intron splicing efficiency and preventing the production of functional proteins, thereby weakening the plant’s defense response.

### 2.5. Identification of the T3SS Effector RipP2 That Regulates AS Using a Splicing Reporter System

The previous phase of research has suggested that T3SS of GMI1000 regulates the AS of specific genes in tomato, thereby influencing plant immunity.

Considering that pre-mRNA splicing takes place within the nucleus of eukaryotic cells, further investigation is aimed at elucidating which specific T3SS effector regulates pre-mRNA splicing and induces AS events that mediate plant immune responses. To this end, the investigation focused on effectors from *R. solanacearum* GMI1000 that localize to the plant nucleus and have characterized functions: RipAB, RipAC, RipAF1, RipN, RipP1, RipP2, and RipX (Table S7).

To identify the effector responsible for AS changes, GFP-tagged versions of these seven nuclear-localized effectors were transiently expressed in transgenic tobacco plants carrying an AS reporter system, *RLPK*-LUC. Confocal microscopy was used to confirm their expression and subcellular localization, and all effectors showed nuclear localization (Figure S3).

**Figure 5:**
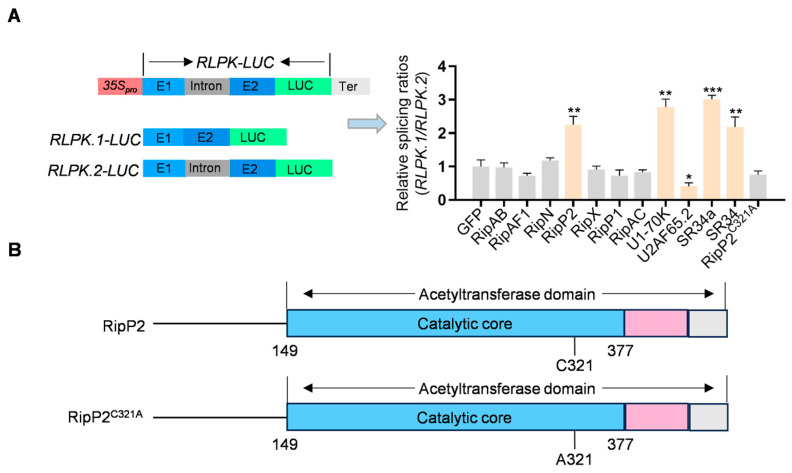
Identification of the T3SS effector RipP2 as a modulator of AS in plants. (**A**) Diagram illustrating the *RLPK-LUC* AS reporter system in transgenic *N. benthamiana*. Among seven nuclear-localized T3SS effectors, only RipP2 causes a significant change in the splicing ratio relative to the EV-GFP control (*p* < 0.01). Statistical significance was assessed using a two-tailed *t*-test: *** *p* < 0.001, ** *p* < 0.01, and * *p* < 0.05. All experiments were performed in triplicate, yielding consistent results. (**B**) Structural diagram of RipP2, illustrating the acetyltransferase domain and the active site at Cys321 (C321). A catalytic mutant, RipP2^C321A^, was generated to abolish acetyltransferase activity. Unlike wild-type RipP2, RipP2^C321A^ did not significantly affect the *RLPK.1/RLPK.2* ratio, indicating that RipP2’s influence on splicing depends on its acetyltransferase function.

This transgenic tobacco line is driven by the 35S promoter to express a 260-bp AS region of the tomato receptor-like protein kinase (*RLPK*) fused to luciferase (LUC), including an intronic region. When AS occurs in this region, the *RLPK.1/RLPK.2* splicing ratio and luciferase activity are regulated (Figure 5A). Previously, this tobacco line has been used in other studies to screen pathogen effectors that induce AS [34,37,46]. To validate the system, four splicing factors—*U1-70K*, *U2AF65C*, *SR34*, and *SR34a*—were expressed, and the *RLPK.1/RLPK.2* splicing ratio was altered, confirming its suitability for validating AS changes (Figure 5A).

The results show that, compared to the GFP control, only the expression of RipP2 significantly altered the *RLPK.1/RLPK.2* splicing ratio, suggesting RipP2 regulates pre-mRNA splicing (Figure 5A).

RipP2, with acetyltransferase activity at Cys321 [47,48], acetylates the WRKY domain of *RRS1-R* in *Arabidopsis*, triggering a hypersensitive response and activating immunity [49]. To further investigate RipP2’s impact on AS, the catalytic mutant RipP2^C321A^ was generated by substituting Cys321 with Ala. Unlike wild-type RipP2, RipP2^C321A^ did not alter the splicing ratio compared to the GFP control (Figure 5A,B), indicating that RipP2 influences splicing through its acetyltransferase activity, playing a role in plant immunity.

### 2.6. Type III Effectors RipP2 Interacts with Splicing Factor SR34a to Manipulate Pre-mRNA Splicing in Planta

Experiments using the *RLPK*-LUC splicing reporter system identified RipP2 as a potential regulator of AS. It is hypothesized that RipP2 might directly or indirectly modulate AS-mediated plant immunity.

Sequence analysis showed no potential RNA-binding domains in RipP2, suggesting it is unlikely to directly interact with pre-mRNA to regulate AS.

Since pre-mRNA splicing occurs within the nucleus of eukaryotic cells and is executed by the spliceosome, it was speculated that the nuclear-localized effector RipP2 might indirectly regulate pre-mRNA splicing by interacting with components of the tomato spliceosome.

**Figure 6:**
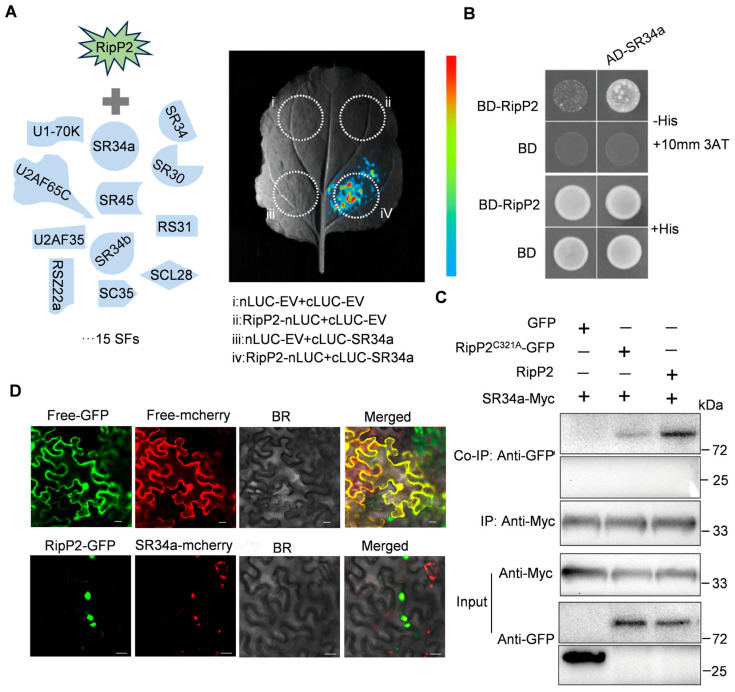
Interaction of RipP2 with splicing factor *SR34a* and its role in modulating host AS. (**A**) Screening of RipP2 interactions with 15 known tomato splicing factors (SFs) using luciferase complementation imaging (LCI) in *N. benthamiana*. Only *SR34a* showed a positive interaction with RipP2, indicated by luminescence (circle iv), absent in the control groups (i–iii). (**B**) Yeast two-hybrid (Y2H) assay supports the specific interaction of RipP2 with *SR34a*. (**C**) Co-immunoprecipitation (Co-IP) in *N. benthamiana* showing that Myc-tagged *SR34a* co-precipitates with GFP-tagged RipP2, but not with GFP alone or the catalytically inactive RipP2^C321A^ mutant, further confirming the specific RipP2-*SR34a* interaction. (**D**) Confocal microscopy of *N. benthamiana* cells showing nuclear co-localizations of RipP2-GFP and *SR34a*-mCherry. All experiments were performed in triplicate, yielding consistent results.

To test this hypothesis, luciferase complementation imaging (LCI) was used to examine the interaction between RipP2 and 15 known tomato splicing factors (Table S7) of the spliceosomal components [34,50,51,52]. The results reveal a specific interaction between RipP2 and the splicing factor *SR34a* (Figure 6A and Figure S4).

Also, yeast two-hybrid (Y2H) assays confirmed the specific binding of RipP2 to *SR34a* (Figure 6B). Yeast co-transformed with BD-RipP2 and AD-*SR34a* grew on the selective medium (-His + 10 mM 3AT), while the control groups showed no growth, ruling out non-specific binding.

These results were further supported by tobacco co-immunoprecipitation (Co-IP) assays, where Myc-tagged *SR34a* co-immunoprecipitated with GFP-tagged RipP2 or its enzymatically inactive point mutant RipP2^C321A^ (Figure 6C).

To examine their subcellular localization, fluorescently tagged fusion proteins of RipP2-GFP and *SR34a*-mCherry were transiently co-expressed in *N. benthamiana*, and their localization was observed using confocal microscopy. RipP2-GFP and *SR34a*-mCherry were found to co-localize in the nucleus, supporting the hypothesis of a functional interaction (Figure 6D).

These observations raised an important question: is the interaction between RipP2 and *SR34a* required for RipP2-mediated AS changes? To address this, the impact of RipP2 on splicing was assessed by silencing *NbSR34a* expression in *RLPK-LUC* transgenic *N. benthamiana* plants by VIGS. In the blank silencing control *RLPK*-*LUC* transgenic *N. benthamiana* leaves, the transient overexpression of RipP2 significantly increased the *RLPK.1/RLPK.2* ratio. However, in Nb*SR34a*-silenced plants, the transient overexpression of RipP2 did not significantly alter the *RLPK.1/RLPK.2* splicing ratio (Figure S4).

In summary, these experiments indicate that the T3SS effector RipP2 from *R. solanacearum* GMI1000 induces AS changes in the host by interacting with the tomato spliceosome core component *SR34a*, which may subsequently modulate the host’s immune response.

### 2.7. RipP2 Acetylates SR34a to Regulate AS and Suppress Plant Immune Responses

RipP2 can interact with the tomato splicing factor *SR34a* in the cell nucleus, and that RipP2 is known to possess acetyltransferase activity, suggesting that RipP2 may acetylate *SR34a* to regulate its function.

**Figure 7:**
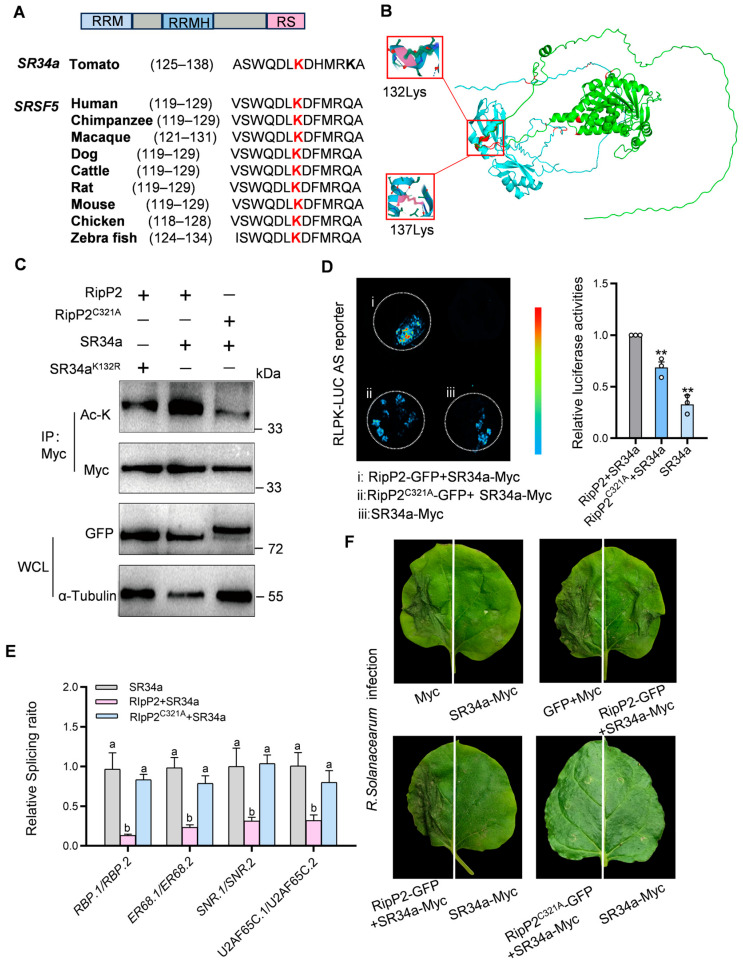
RipP2-mediated acetylation of *SR34a* modulates splicing and plant immunity. (**A**) Domain structure and conserved motif alignment of *SR34a* in various species, highlighting the SWQDLKD motif with Lys132. (**B**) AlphaFold2-predicted 3D structure of the RipP2-*SR34a* complex. RipP2 is shown in green, while *SR34a* is shown in blue. The key interaction sites, including K132 and K137 in *SR34a*, are highlighted. (**C**) In vivo acetylation assay. Co-expression of RipP2 and *SR34a* in *N. benthamiana* leaves resulted in *SR34a* acetylation. This acetylation was reduced when RipP2^C321A^ or *SR34a*^K132R^mutants were co-expressed, suggesting that RipP2 acetylates *SR34a* at Lys132. (**D**) *RLPK*-LUC AS reporter assay in transgenic *N. benthamiana* shows that the RipP2-mediated acetylation of *SR34a* significantly increases *RLPK*.1 isoform levels compared to RipP2^C321A^or *SR34a* alone. Statistical significance was assessed using a two-tailed *t*-test: ** *p* < 0.01. (**E**) Quantification of splicing ratios for four immune-related genes (*RBP*, *ER68*, *SNR*, and *U2AF65C*) in tomato. Different letters indicate statistically significant differences (*p* < 0.05, one-way ANOVA followed by Duncan’s multiple range test). (**F**) Disease resistance assay in *N. benthamiana*. *SR34a* expression enhances resistance to *R. solanacearum*, while co-expression with RipP2 negates this effect. In contrast, RipP2^C321A^co-expression with *SR34a* maintains resistance, supporting the role of RipP2-mediated acetylation in modulating *SR34a*’s immune function. All experiments were performed in triplicate, yielding consistent results.

*SR34a* belongs to the SR subfamily of plant SR proteins and exhibits typical SR protein characteristics, including two RNA recognition motifs (RRMs), an RRM homolog (RRMH), and a C-terminal domain rich in arginine and serine (RS) dipeptide repeats. In most SR proteins with two RNA-binding domains, the RRMH contains an evolutionarily conserved SWQDLKD motif [53,54,55,56,57]. Figure 7A shows the conserved SWQDLKD motif in SR proteins of different species (Figure 7A). Mass spectrometry studies have shown that, in mammals, the lysine(K) residue at position 125(K125) within the SWQDLKD motif is an acetylation site for the Tip60 acetyltransferase and is also a target for ubiquitination [13,58]. Based on this, it is hypothesized that the conserved K residue within the SWQDLKD motif in plant SR proteins may similarly serve as an acetylation site.

Sequence analysis revealed that in *SR34a*, this conserved K residue in the SWQDLKD motif is located at amino acid position 132(K132).

Interestingly, the 3D structure of the RipP2-*SR34a* complex, predicted using AlphaFold2, revealed a high confidence score of 0.9604. In this structure, RipP2 (green) interacts with *SR34a* (blue), with the α-helix region of *SR34a* (residues 89–141) forming a flexible, surface-exposed structure that binds to RipP2 (residues 111–159). Key residues K132 and K137 in *SR34a*’s SWQDLKDHMRK motif were identified as critical interaction sites, and it is proposed that K132 serves as the primary acetylation site (Figure 7B and Figure S5).

To test this, the K132 in *SR34a* was mutated to R (*SR34a*K132R-Myc) and an in vivo acetylation assay was performed. Co-expression of RipP2 and *SR34a* in *N. benthamiana* showed the acetylation of *SR34a*, which was markedly reduced with the co-expression of RipP2^C321A^ and *SR34a* or RipP2 and *SR34a*K132R, indicating that RipP2 acetylates *SR34a* at K132. Western blot analysis confirmed consistent protein expression (Figure 7C), and LCI showed a weakened RipP2-*SR34a* interaction with the K132 mutation (Figure S5). These results confirm that RipP2 acetylates *SR34a* primarily at K132.

To investigate the impact of RipP2-mediated *SR34a* acetylation on pre-mRNA AS, RipP2-GFP was co-expressed with *SR34a*-Myc, RipP2^C321A^-GFP was co-expressed with *SR34a*-Myc, and *SR34a*-Myc was expressed alone in RLPK-LUC transgenic *N. benthamiana*. Luciferase activity was the highest in the RipP2-GFP and *SR34a* co-expression group (Figure 7D). Activity in the RipP2^C321A^-GFP and *SR34a* group was 69%, and in the *SR34a*-only group, 33% of the RipP2-GFP and *SR34a* co-expression group, indicating that RipP2 acetylates *SR34a* to promote intron splicing in the tomato RLPK gene.

To further validate the regulatory effect of RipP2-acetylated *SR34a* on the splicing of tomato defense-related genes, *SR34a* alone, RipP2 with *SR34a*, and RipP2^C321A^ with *SR34a* were overexpressed in tomato, and the splicing ratios of four positive immune regulatory genes (*RBP*, *ER68*, *SNR*, and *U2AF65C*) in the spliceosome pathway were measured. The results indicate that, compared to *SR34a* overexpression alone, the co-expression of RipP2 and *SR34a* significantly reduced the proportion of functional splice isoforms, suggesting that RipP2 inhibits the effect of *SR34a* on the intron splicing of these genes. However, the co-expression of RipP2^C321A^ with *SR34a* yielded splicing ratios similar to the *SR34a*-only group, indicating no significant difference (Figure 7E) and demonstrating that the acetyltransferase activity of RipP2 inhibits the intronic splicing of these four immune positive regulators by *SR34a*.

The interaction between RipP2 and *SR34a* has a dual regulatory effect on tomato splicing: RipP2-*SR34a* promotes intron splicing in the RLPK gene, increasing intron-spliced functional isoform RLPK.1, while suppressing splicing in four immune-related genes (*RBP*, *ER68*, *SNR*, and *U2AF65C*), reducing intron-spliced functional isoforms. This mechanism highlights how RipP2 selectively modulates the splicing of defense genes by acetylating *SR34a*, providing insights into how pathogens disrupt host splicing to weaken immunity.

To further verify the role of RipP2-mediated *SR34a* acetylation in regulating plant immunity, a final set of disease resistance assays was conducted. *N. benthamiana* leaves transiently co-expressing different proteins were inoculated with the *R. solanacearum* pathogen, and lesion size and plant wilting severity were observed. The results show that *SR34a* expression alone significantly enhances disease resistance compared with the control, as evidenced by smaller lesion areas and milder wilting symptoms (Figure 7F). However, in the group co-expressing RipP2 and *SR34a*, disease symptoms were similar to the control and significantly reduced compared to the resistance of *SR34a* alone. In contrast, leaves co-expressing RipP2^C321A^ with *SR34a* showed enhanced resistance similar to the *SR34a*-only group. These data support that the acetyltransferase activity of RipP2 affects the role of *SR34a* in plant immunity by regulating its splicing function.

These findings suggest that the T3SS effector RipP2 from *R. solanacearum* GMI1000 regulates host AS by acetylating the lysine (K132) residue in the SWQDLKD motif of the tomato spliceosome component *SR34a*. RipP2’s acetyltransferase activity modulates *SR34a*-mediated intron splicing of immune-related genes, particularly inhibiting the intron splicing of four immune positive regulators within the spliceosome pathway, thereby weakening plant disease resistance and disrupting immune responses. These results provide new insights into how pathogens manipulate the host splicing pathway to impair defense mechanisms and lay the foundation for further research on RipP2 and its acetylation targets in plant pathogenesis.

## 3. Discussion

In this study, the role of T3SS of the *R. solanacearum* model strain GMI1000 in the regulation of tomato gene splicing was investigated. It was found that the T3SS effector protein RipP2 regulates defense-related genes AS by acetylating *SR34a*, thereby affecting plant immune responses. T3SS modulates the splicing patterns of immune genes through RipP2, particularly four genes associated with immune responses—*RBP* (RNA binding protein), *SNR* (small nuclear ribonucleoprotein), *ER68* (ethylene-responsive RNA helicase), and *U2AF65C* (U2AF large subunit)—which promote intron retention (IR). This phenomenon may lead to the production of truncated, non-functional proteins, ultimately suppressing immune responses and reducing plant resistance to *R. solanacearum*.

**Figure 8:**
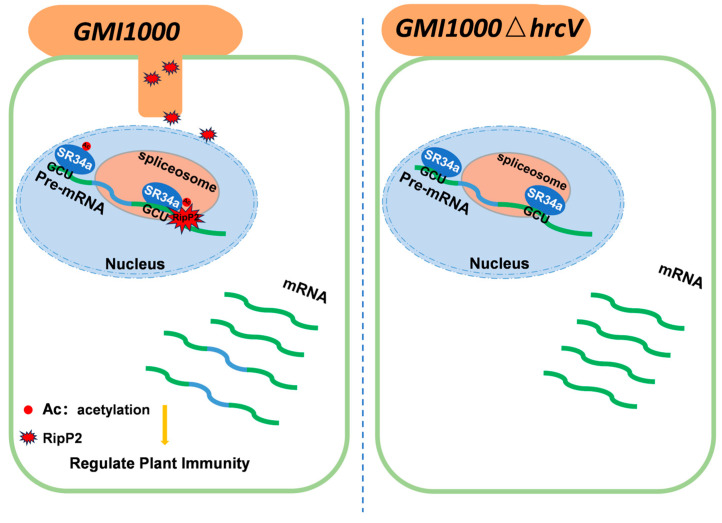
Working model of the RipP2-mediated regulation of *SR34a* acetylation and splicing activity in plant immunity.

The four defense-related genes identified within the spliceosome pathway (*RBP*, *ER68*, *SNR*, and *U2AF65C*) exhibit characteristics of intron retention, suggesting that splicing changes in these genes could influence the plant’s immune response and alter its resistance to pathogens. This process is further explained by the proposed working model in Figure 8, where RipP2’s acetylation of *SR34a* is a key step in regulating immune gene splicing. The absence of splicing regulation in the RipP2^C321A^ mutant further underscores the importance of RipP2’s acetyltransferase activity in immune regulation.

RipP2 is the first avirulence effector identified in *R. solanacearum*, and considerable research has been conducted on its function. Previous studies revealed its interaction with the *A. thaliana* R gene RRS1-R [59] and the cysteine protease RD19 [60], which triggers plant immunity. Further research indicated that the WRKY domain of RRS1 in the RRS1/RPS4 complex acts as a target for RipP2 acetylation, preventing RipP2 from interfering with WRKY transcription factors and converting RipP2’s lysine acetyltransferase activity into an immune response [49]. The research on *SR34a* also has mainly focused on *Arabidopsis*, where *SR34a* primarily binds to exonic sequences rich in GCU near splice sites and tends to target pre-mRNA of ABA-sensitive genes to prevent ABA-responsive splicing in germinating seeds [61,62,63].

The findings of this work add to the existing research by revealing a novel mechanism of RipP2 in plant immunity, demonstrating that RipP2 regulates host splicing events through the acetylation of *SR34a*, thereby influencing the function of immune genes.

Although previous studies have shown that several pathogen effectors promote infection by modulating host splicing events, the role of RipP2 in regulating AS through the acetylation of *SR34a* in plant immunity is a novel discovery. This not only expands the understanding of the functional roles of T3SS effectors, but also provides new insights into exploring novel mechanisms of plant immune regulation.

In addition to its role in immunity, it is proposed that the RipP2-*SR34a* module may also regulate non-immune pathways, particularly metabolism and development. RNA-seq data from *R. solanacearum* GMI1000 and the *hrcV* mutant strain reveal significant splicing changes in genes related to both metabolism and development under infection conditions. For example, the *GPCR* (G protein-coupled receptor) gene may mediate the transmission of environmental signals and regulate metabolic pathways, while the *CFP* (cyclin family protein) gene is crucial for cell cycle progression and tissue development. Furthermore, the *SMSF* (motor neuron splicing factor) may influence developmental processes by modulating the splicing of key genes involved in cell fate and differentiation. These findings suggest that the splicing regulatory role of *SR34a* may be broader than previously thought, affecting not only immune-related genes but also genes involved in fundamental physiological processes, such as metabolism and development.

However, there are limitations to this study. Due to the challenges in expressing tomato genes, some experiments were shifted to tobacco plants, which may introduce species-specific differences. Additionally, while RNA-seq revealed splicing pattern changes, it did not fully capture all splicing events of relevant genes, potentially due to sequencing depth and sample handling. Future research should further explore the role of RipP2 in spliceosome assembly and, by integrating more high-throughput techniques, continue to uncover the multifaceted impacts of T3SS on plant immunity and splicing regulation.

## 4. Materials and Methods

### 4.1. Plant and Microbial Culture Conditions

The tomato plants (*Solanum lycopersicum*, cv. Heinz 1706) were cultivated in a greenhouse under conditions of 24 °C for a 14 h light period and 22 °C for a 10 h dark period for 8–10 weeks. The *N.benthamiana* plants were grown in a greenhouse at 22 °C with a 16 h light period and an 8 h dark period for 5–6 weeks.

*R. solanacearum* strains GMI1000 and CQPS-1 were obtained from CIRM-CFBP, France, and isolated and provided by the Laboratory of Natural Products Pesticides, College of Plant Protection, Southwest University, Chongqing, China, respectively. These strains were revived on solid GB medium for 2 days, followed by regular cultivation in liquid GB medium at 28 °C.

*Agrobacterium tumefaciens* GV3101 strains, each carrying different plasmids, were cultured at 28 °C in LB medium supplemented with 25 mg/mL of rifampicin and 50 mg/mL of kanamycin.

### 4.2. N. benthamiana and Tomato Inoculation Assay

Pathogen inoculation assays were conducted using *R. solanacearum*. The roots of sterile-grown tomato seedlings were immersed in an *R. solanacearum* suspension with an OD600 = 1.0, ensuring the complete immersion of the roots. The roots were soaked for 5–10 min, after which the seedlings were transplanted into 90 mm Petri dishes containing hydroponic medium and incubated at 28 °C [64].

The wounded roots of 4-week-old *Arabidopsis* plants were inoculated with 15 mL of an *R. solanacearum* suspension with an OD600 = 0.1 using the soil drenching inoculation method. The plants were placed in a growth chamber under controlled conditions of 75% humidity, a 12 h light/12 h dark photoperiod, and incubated at 28 °C. Plant disease symptoms were evaluated based on the following scale: 0: no wilting; 1: 1–25% wilting; 2: 26–50% wilting; 3: 51–75% wilting; and 4: 76–100% wilting.

Bacterial quantification of the roots of both *Arabidopsis* and tomato plants was performed at 3 and 5 days post-inoculation (dpi). Roots from six independent plants were harvested, weighed, and subjected to bacterial quantification. Subsequently, the root tissues were ground in 100 mL of ddH_2_O, and 10 mL of the diluted solution was plated onto solid GB medium for bacterial enumeration.

For the transient gene expression assay, specific gene fragments were cloned into the vectors for overexpression using pDT1-myc and pDT7-GFP vectors. *A. tumefaciens* strain GV3101 carrying pDT1-myc or pDT7-GFP constructs was cultured to an OD = 0.5 *A. tumefaciens* cultures were resuspended in infiltration buffer (10 mM MgCl2, 10 mM MES [pH 5.6], and 150 mM acetosyringone) to a final concentration of OD600 = 0.5. The resulting suspension was infiltrated into plant leaves to achieve the transient expression of the gene. Fusion protein expression was detected using immunoprecipitation blotting or confocal laser scanning microscopy (Zeiss LSM 880, Carl Zeiss AG, Oberkochen, Germany) to detect green fluorescence and mCherry.

For the VIGS assay, TRV1 and TRV2 vectors were used, with specific gene fragments cloned into the TRV2 vector to target genes of interest. The TRV2-PDS construct served as a positive control, targeting the phytoene desaturase (PDS) gene to induce bleaching, alongside TRV2-*RBP*, TRV2-*ER68*, TRV2-*SNR*, and TRV2-*U2AF65C* to investigate their functions. *A. tumefaciens* strain GV3101 carrying TRV1 and TRV2 constructs was cultured to an OD = 0.5. *Agrobacterium* suspensions of TRV1 and TRV2 were mixed at a 1:1 ratio and infiltrated into tomato leaves. Plants were maintained under controlled conditions, and gene silencing efficiency was assessed by RT-qPCR 7 days post-infiltration. Pathogen inoculation was subsequently performed using the soil drenching method.

### 4.3. R. solanacearum Knockout Mutants

Homologous recombination was used to replace the *hrcV* and *hrpB* coding sequences with a kanamycin resistance gene, generating the *R. solanacearum* T3SS mutant. PCR amplification was performed to obtain the upstream (UP) and downstream (DN) regions flanking the *hrcV* or *hrpB* gene and the kanamycin resistance gene fragment. These regions were fused with the kanamycin resistance gene. The resulting UP-KAN-DN fusion fragment was introduced into the competent *R. solanacearum* GMI1000 strain by electroporation. Mutant strains were selected using kanamycin (25 mg/mL) and confirmed by PCR with specific primers for *hrcV*, *hrpB*, and the *R. solanacearum* flagellin gene [65].

### 4.4. Co-IP Assays

The target gene PCR fragments were ligated into the linearized vectors pDT7-GFP and pDT1-Myc. The constructs pDT7-GFP-RipP2/RipP2C321A and pDT1-Myc-SR34a were transiently expressed in *N. benthamiana* leaves via *A. tumefaciens* GV3101 infiltration.

After 48 h, the leaves were ground in liquid nitrogen, and total protein was extracted with Beyotime RIPA lysis buffer (5 mL/g tissue, with protease inhibitors) at 4 °C for 30 min. The lysate was centrifuged at 12,000 rpm for 10 min to collect the supernatant. For immunoprecipitation, 500 μL of protein extract (500–1000 μg) was incubated with 20 μL of anti-Myc magnetic beads at 4 °C for 12 h, followed by four washes with RIPA buffer. Proteins were eluted with 2× SDS buffer and heated at 95 °C for 5 min. Samples were separated by SDS-PAGE (Bio-Rad, Hercules, CA, USA), transferred to PVDF(Millipore, Burlington, MA, USA) membranes, probed with anti-GFP and anti-Myc antibodies, and visualized by enhanced chemiluminescence (ECL). GFP alone was expressed as a control for interaction specificity.

### 4.5. RNA-seq and Data Analysis

RNA-Seq Sample Collection: Seven-day-old tomato seedlings were inoculated as described in the infection assay and incubated at 28 °C. Samples were harvested 72 h post-inoculation and immediately flash-frozen in liquid nitrogen. Total RNA was extracted using the Plant Total RNA Isolation Kit (B518631-0050; Sangon Biotech, Shanghai, China) according to the manufacturer’s protocol, and RNA-seq libraries were constructed. Sequencing was performed on the Illumina HiSeq platform in paired-end mode with a read length of 150 bp (Sangon Biotech, Shanghai, China).

Raw sequencing data were filtered using Trimmomatic to obtain high-quality clean reads. Quality-filtered reads were then aligned to the ITAG2.4 tomato reference genome using HISAT2, with alignment quality assessed using RSeQC. Mapped reads from all datasets were assembled using StringTie, and gene expression levels were quantified based on known gene models. Differential expression analysis was performed with DESeq2, and the results were visualized to facilitate downstream analysis.

Differential alternative splicing (DAS) events were identified using rMATS 4.1.1, including five splicing types: ES (exon skipping), A5SS (alternative 5′ splice site), A3SS (alternative 3′ splice site), MEXs (mutually exclusive exons), and IR (intron retention). The parameters used were: rmats.py --b1 B.txt --b2 A.txt --gtf genes.gtf --od B_vs_A -t paired --readLength 150 --nthread 10 --tmp test/ --novelSS. Events with a *p*-value < 0.05 were considered as significantly differential AS events. PSI (Percent Spliced In) was calculated using rMATS [44], representing the percentage of the inclusion isoform (long variant), defined as the ratio of the inclusion isoform (splice-in) to the sum of the inclusion isoform (splice in) and the exon exclusion isoform (splice out).

### 4.6. DAS Event Visualization and RT-qPCR Analysis

RNA-seq data were visualized using Integrative Genomics Viewer (IGV) version 2.11.9, which displayed the read coverage of exonic and intronic regions of the target tomato genes in wiggle plots. The software was used to analyze the splicing events of these genes.

Total RNA from tomato was extracted using the Plant Total RNA Isolation Kit (B518631-0050; Sangon Biotech, Shanghai, China). cDNA was synthesized using the HiScript IV RT SuperMix for qPCR (R423-01; Vazyme Biotech Co., Ltd., Nanjing, China). Isoform-specific primers were designed to validate DAS events based on regions of intron retention and exon skipping. The splicing ratios of *RLPK*, *RBP*, *SP1*, *ER68*, *SR45a*, *U2AF65C*, *GPCR*, *SNR*, *CFP*, and *SMSF* were calculated as relative values, with the splicing ratio defined as the ratio of the spliced isoform to the isoform containing either exons or introns.

The expression of *gyrA* was used as an internal reference to normalize the expression levels of effector genes in *R. solanacearum*. The expressions of *SlUBI* and *NbEF1α* were used as the internal references to normalize the expression level of genes in tomato and *N. benthamiana* plants, respectively.

### 4.7. Luciferase Complementation Assay

Luciferase complementation assays were performed as described [66]. The RipP2 gene was inserted into the pCAMBIA1300-nLUC vector, while 15 splicing factor genes were inserted into the pCAMBIA1300-cLUC vector. *Agrobacterium* suspensions containing these constructs were infiltrated into 6-week-old *N. benthamiana* leaves. After 48 h of infiltration, the leaves were collected, and for LUC imaging, 1 mM of luciferin substrate was applied. LUC images were captured using the Tanon-5200 Multi Chemiluminescent Imaging System (Tanon, China). For the quantification of LUC signals from the luciferase complementation assay and the *RLPK*-LUC reporter system, ImageJ software (version 1.53c) was utilized.

### 4.8. Yeast Two-Hybrid Assay for the RipP2-SR34a Interaction

A yeast two-hybrid assay was conducted to assess the interaction between RipP2 and *SR34a*. The coding sequences of RipP2 and *SR34a* were cloned into pGBKT7 and pGADT7 vectors to generate BD-RipP2 and AD-*SR34a* constructs. Yeast strain Y2HGold was co-transformed with BD-RipP2 and AD-*SR34a*, along with controls (BD with AD-*SR34a*, BD-RipP2 with AD). Transformants were plated on selective medium (-His) supplemented with 0–20 mM 3AT to assess interaction specificity and on non-selective medium (+His) to confirm viability and transformation efficiency. Growth on the -His medium with 3AT indicated a specific interaction between RipP2 and *SR34a*.

### 4.9. Statistical Analysis

Statistical analyses were performed using GraphPad Prism 5 software (GraphPad Software, Boston, MA, USA). In all cases, a value of *p* < 0.05 was considered statistically significant.

### 4.10. Accession Numbers

The sequence data and functional information of the *R. solanacearum* Type III secretion system effectors presented in this study can be accessed through the NCBI database and the Nemo Peeters *R. solanacearum* Type III Effectors Database. The gene sequence data of Heinz 1706 tomato referenced in this study can be accessed through the Sol Genomics Network (https://solgenomics.sgn.cornell.edu/ (accessed on 1 December 2024). The RNA-seq data that support the findings of this study have been deposited in the Gene Expression Omnibus (GEO) database (https://www.ncbi.nlm.nih.gov/geo/ (accessed on 1 December 2024) with the accession code GEO: GSE276633.

## 5. Conclusions

In this study, it is demonstrated that *R. solanacearum* GMI1000 employs its T3SS effector RipP2 to manipulate genome-wide alternative splicing in tomato. RipP2 interacts with and acetylates the splicing factor *SR34a* at a conserved lysine residue at position 132, altering its splicing pattern on defense-related genes, particularly those in the spliceosome pathway, such as *RBP*, *ER68*, *SNR*, and *U2AF65C*. This modification disrupts the splicing of defense-associated transcripts, undermining plant immunity. These findings reveal a previously uncharacterized mechanism by which a bacterial effectors targets host splicing machinery to modulate gene expression and facilitate infection. This discovery provides key molecular targets for understanding how pathogens suppress immune functions by altering the host splicing machinery. These targets hold significant potential for future applications in disease resistance breeding and immune regulation research. This work advances the understanding of pathogen–host interactions and highlights the critical role of alternative splicing regulation in plant defense.

## Data Availability

Data underlying the results of this study can be obtained from the corresponding authors upon request.

## Supplementary Materials

The following supporting information can be downloaded at: https://www.mdpi.com/article/10.3390/plants14040534/s1, Figure S1: RNA-seq mapping statistics and sample correlations for tomato plants inoculated with GMI1000 and △hrcV. (a) Summary of RNA-seq read mapping results. A total of 257,709,242 reads were mapped to the Solanum lycopersicum genome, with detailed statistics on total mapped reads, uniquely mapped reads, splice reads, and proper pair alignments across six samples (three biological replicates per treatment). (b) Heatmap of Pearson correlation coefficients among samples. Biological replicates of △hrcV (hrcV-1, hrcV-2, hrcV-3) and GMI1000 (GMI1000-1, GMI1000-2, GMI1000-3) form distinct clusters, reflecting high intra-group consistency and treatment-specific transcriptomic differences. These results validate the RNA-seq data quality and support downstream analyses of T3SS-mediated alternative splicing regulation. Table S1: Statistics of alternative splicing events. Table S2: Differentially Expressed Genes (DEGs) and Differentially Alternatively Spliced Genes (DASGs). Table S3: The proportion of each splicing type in differential and non-differential alternative splicing events. Table S4: Significant_Splicing_Changes___PSI___0_05_. Table S5: rMATS.gene_KEGG_enrichment. Tbale S6: The Nucleic Acid Sequence of Different Isoforms Described in Figure 4. Table S7: The sequences of seven subcellular nuclear localization type III secretion system effectors and the Sequences of 15 Splicing Factors. Table S8: List of Primers Used in This Study, Related to Methods.
