# Supplementary material for: A Ralstonia solanacearum Effector Targets Splicing Factor SR34a to Reprogram Alternative Splicing and Regulate Plant Immunity
Source: Plants (Basel). 2025 Feb 10;14(4):534. doi: 10.3390/plants14040534 (PMC11859261; doi:10.3390/plants14040534)
Supplement: Supplementary file 1 [file plants-14-00534-s001.zip › Supplemental Figure.pdf]

Supplemental Figure

(a)

	hrcV-1	hrcV-2	hrcV-3	GMI1000-1	GMI1000-2	GMI1000-3
Total reads	37616868 (100.00%)	37452744 (100.00%)	44085682 (100.00%)	40998448 (100.00%)	39993072 (100.00%)	39993072 (100.01%)
Total mapped	34874663 (92.71%)	34769563 (92.84%)	40941717 (92.87%)	28024859 (68.36%)	28273876 (70.70%)	28273876 (70.71%)
Mutiple mapped	531369 (1.41%)	532922 (1.42%)	620156 (1.41%)	378411 (0.92%)	390869 (0.98%)	390869 (0.99%)
Uniquely mapped	34343294 (91.30%)	34236641 (91.41%)	40321561 (91.46%)	27646448 (67.43%)	27883007 (69.72%)	27883007 (69.73%)
Read-1 mapped	17246957 (45.85%)	17195284 (45.91%)	20239043 (45.91%)	13873651 (33.84%)	14004256 (35.02%)	14004256 (35.03%)
Read-2 mapped	17096337 (45.45%)	17041357 (45.50%)	20082518 (45.55%)	13772797 (33.59%)	13878751(34.70 %)	13878751 (34.71%)
Reads map to '+'	17156642 (45.61%)	17102635 (45.66%)	20142407 (45.69%)	13816709 (33.70%)	13932376 (34.84%)	13932376(34.85 %)
Reads map to '-'	17186652 (45.69%)	17134006 (45.75%)	20179154 (45.77%)	13829739 (33.73%)	13950631 (34.88%)	13950631 (34.89%)
Non-splice reads	21615493 (57.46%)	21585838 (57.63%)	25683751(58.26 %)	18964830 (46.26%)	18622150 (46.56%)	18622150 (46.57%)
Splice reads	12727801 (33.84%)	12650803 (33.78%)	14637810 (33.20%)	8681618 (21.18%)	9260857 (23.16%)	9260857 (23.17%)
Reads mapped in proper pairs	33758246 (89.74%)	33640926 (89.82%)	39625470 (89.88%)	27230676 (66.42%)	27440778 (68.61%)	27440778 (68.62%)

(b)

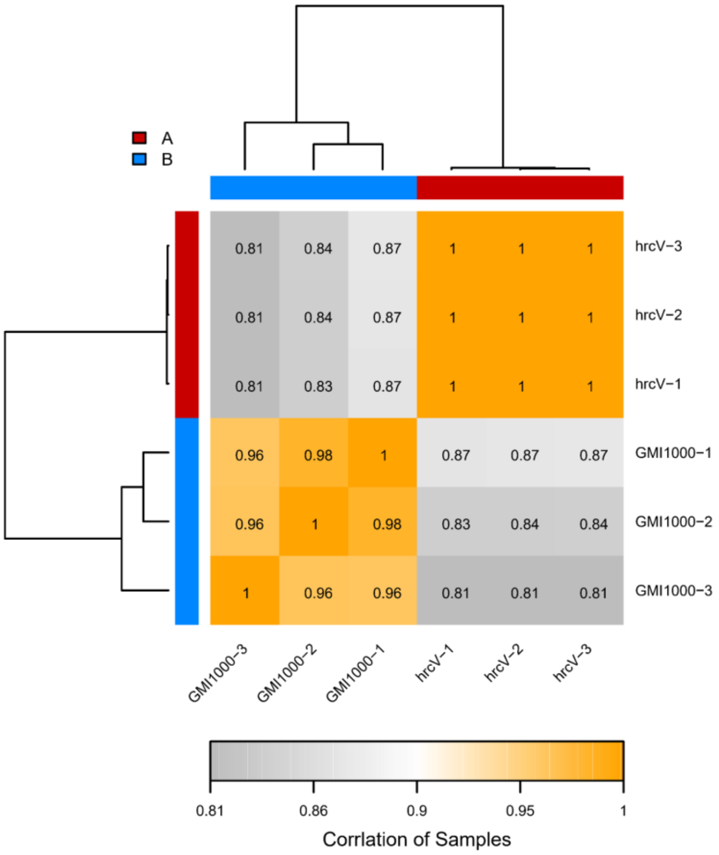

Figure S1. RNA-seq mapping statistics and sample correlations for tomato plants inoculated with GMI1000 and △hrcV.

(a) Summary of RNA-seq read mapping results. A total of 257,709,242 reads were mapped to the *Solanum lycopersicum* genome, with detailed statistics on total mapped reads, uniquely mapped reads, splice reads, and proper pair alignments across six samples (three biological replicates per treatment).

(b) Heatmap of Pearson correlation coefficients among samples. Biological replicates of △*hrcV* (hrcV-1, hrcV-2, hrcV-3) and GMI1000 (GMI1000-1, GMI1000-2, GMI1000-3) form distinct clusters, reflecting high intra-group consistency and treatment-specific transcriptomic differences.

These results validate the RNA-seq data quality and support downstream analyses of T3SS-mediated alternative splicing regulation.

(a)

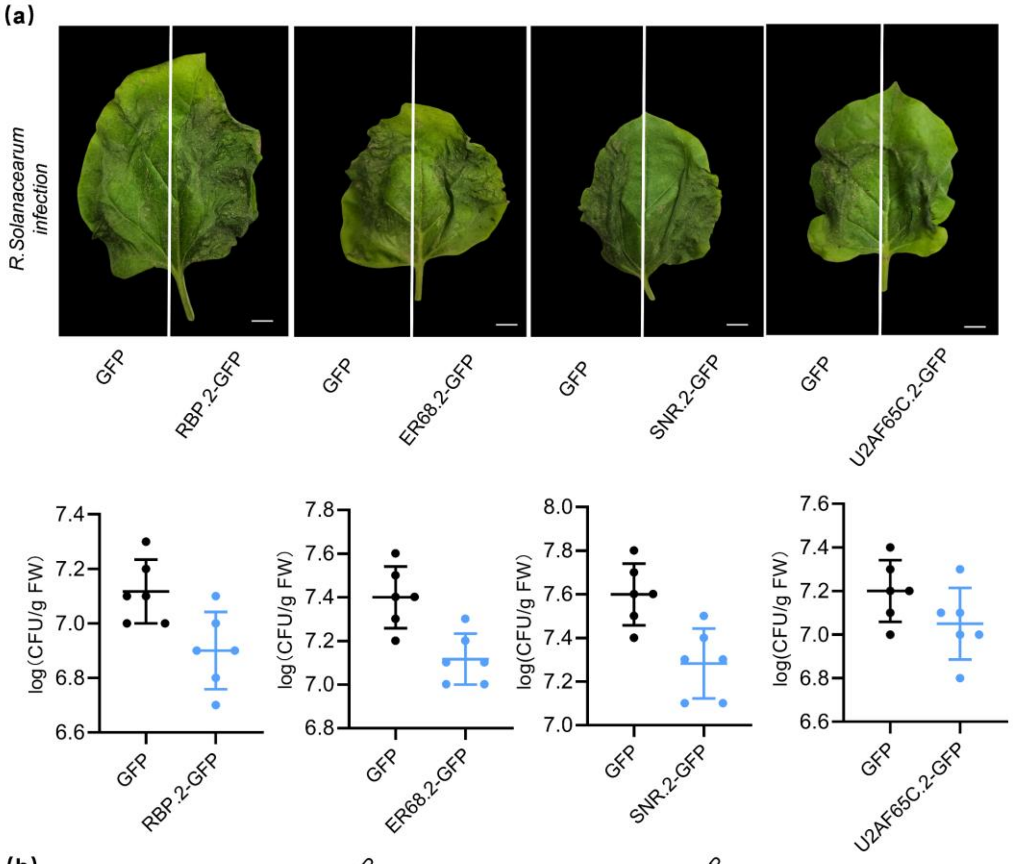
R.Solanacearum infection
GFP
RBP.2-GFP
GFP
ER68.2-GFP
GFP
SNR.2-GFP
GFP
U2AF65C.2-GFP
log(CFU/g FW)
7.4
7.2
7.0
6.8
6.6
GFP
RBP.2-GFP
7.8
7.6
7.4
7.2
7.0
6.8
GFP
ER68.2-GFP
8.0
7.8
7.6
7.4
7.2
7.0
GFP
SNR.2-GFP
7.6
7.4
7.2
7.0
6.8
6.6
GFP
U2AF65C.2-GFP

(b)

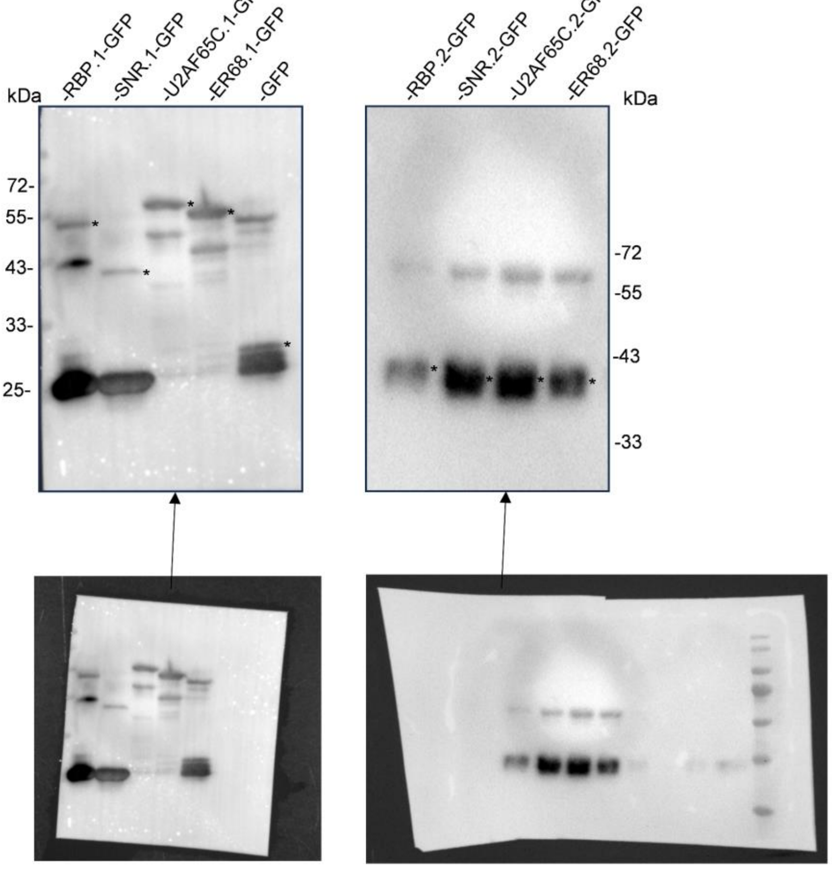
-RBP.1-GFP
-SNR.1-GFP
-U2AF65C.1-GFP
-ER68.1-GFP
-GFP
kDa
72-
55-
43-
33-
25-
-RBP.2-GFP
-SNR.2-GFP
-U2AF65C.2-GFP
-ER68.2-GFP
kDa
-72
-55
-43
-33

Figure S2. Functional validation of GFP-tagged splice isoforms in *Nicotiana benthamiana* during *Ralstonia solanacearum* infection.
(a) Bacterial growth assays in leaves expressing GFP-tagged isoforms. Functional isoforms (*RBP.1, ER68.1, SNR.1, and U2AF65C.1*) significantly reduced bacterial growth compared to the GFP control, while non-functional isoforms *(RBP.2, ER68.2, SNR.2, and U2AF65C.2*) had no effect.
(b) Immunoblot analysis confirming protein expression. Functional isoforms produced full-length proteins, whereas non-functional isoforms yielded truncated or unstable products.
These data reveal the functional divergence of splice isoforms in plant immunity.

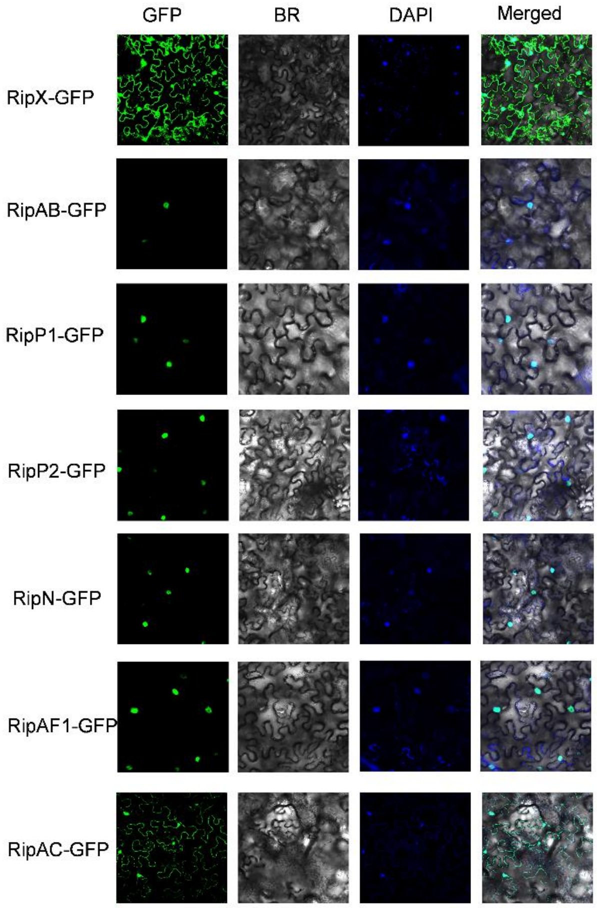

Figure S3. Nuclear localization of GFP-tagged T3SS effectors from ***Ralstonia solanacearum***.

Seven T3SS effectors (RipX, RipAB, RipP1, RipP2, RipN, RipAF1, and RipAC) were transiently expressed in *Nicotiana benthamiana*. Confocal microscopy, performed using a Zeiss LSM 880 system, confirmed their nuclear localization, as indicated by GFP signals colocalizing with DAPI-stained nuclei.

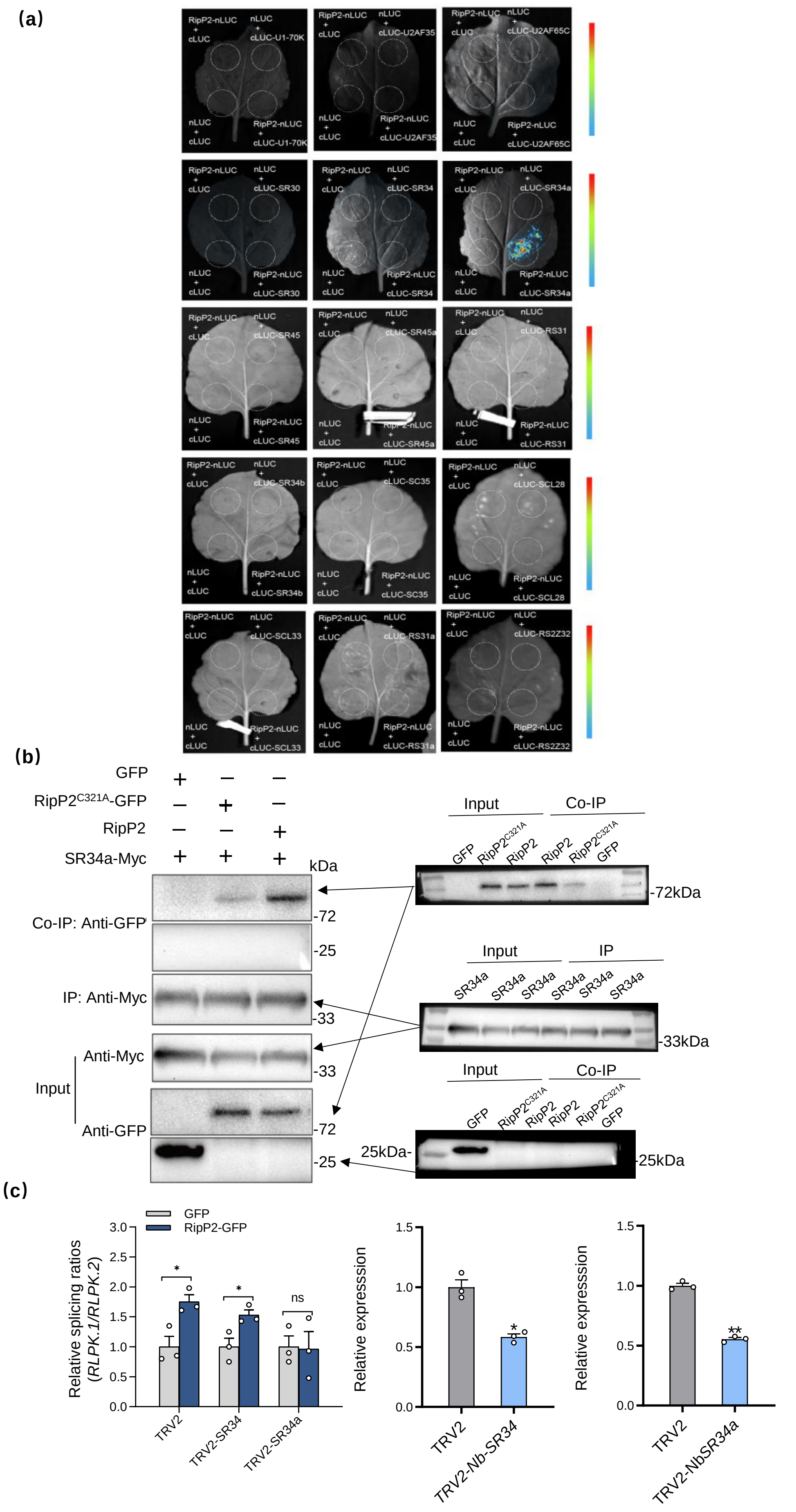
(a)
RipP2-nLUC + cLUC
nLUC + cLUC-U1-70K
nLUC + cLUC
RipP2-nLUC + cLUC-U1-70K
nLUC + cLUC-U2AF35
RipP2-nLUC + cLUC-U2AF35
nLUC + cLUC-U2AF65C
RipP2-nLUC + cLUC-U2AF65C
nLUC + cLUC-SR30
RipP2-nLUC + cLUC-SR30
nLUC + cLUC-SR34
RipP2-nLUC + cLUC-SR34
nLUC + cLUC-SR34a
RipP2-nLUC + cLUC-SR34a
nLUC + cLUC-SR45
RipP2-nLUC + cLUC-SR45
nLUC + cLUC-SR45a
RipP2-nLUC + cLUC-SR45a
nLUC + cLUC-RS31
RipP2-nLUC + cLUC-RS31
nLUC + cLUC-SR34b
RipP2-nLUC + cLUC-SR34b
nLUC + cLUC-SC35
RipP2-nLUC + cLUC-SC35
nLUC + cLUC-SCL28
RipP2-nLUC + cLUC-SCL28
nLUC + cLUC-SCL33
RipP2-nLUC + cLUC-SCL33
nLUC + cLUC-RS31a
RipP2-nLUC + cLUC-RS31a
nLUC + cLUC-RS2Z32
RipP2-nLUC + cLUC-RS2Z32
(b)
GFP + – –
RipP2C321A-GFP – + –
RipP2 – – +
SR34a-Myc + + +
kDa
Co-IP: Anti-GFP
-72
-25
IP: Anti-Myc
-33
Input
Anti-Myc
-33
Anti-GFP
-72
-25
Input
Co-IP
GFP RipP2C321A RipP2 RipP2 RipP2C321A GFP
-72kDa
Input
IP
SR34a SR34a SR34a SR34a SR34a SR34a
-33kDa
Input
Co-IP
GFP RipP2C321A RipP2 RipP2 RipP2C321A GFP
25kDa-
-25kDa
(c)
GFP
RipP2-GFP
Relative splicing ratios (RLPK.1/RLPK.2)
3.0
2.5
2.0
1.5
1.0
0.5
0.0
*
*
ns
TRV2
TRV2-SR34
TRV2-SR34a
Relative expression
1.5
1.0
0.5
0.0
*
TRV2
TRV2-Nb-SR34
Relative expression
1.5
1.0
0.5
0.0
**
TRV2
TRV2-NbSR34a

Figure S4. RipP2 interacts with SR34a to regulate alternative splicing in *Nicotiana benthamiana*.
(a) LCI assay showing a specific interaction between RipP2 and SR34a, with no detectable interactions between RipP2 and other tested splicing factors.
(b) Co-immunoprecipitation (Co-IP) assay confirming the interaction between GFP-tagged RipP2 and Myc-tagged SR34a.
(c) Silencing of NbSR34a abolishes RipP2-mediated changes in the RLPK.1/RLPK.2 splicing ratio in RLPK-LUC transgenic plants, indicating that SR34a is required for RipP2-induced splicing regulation.

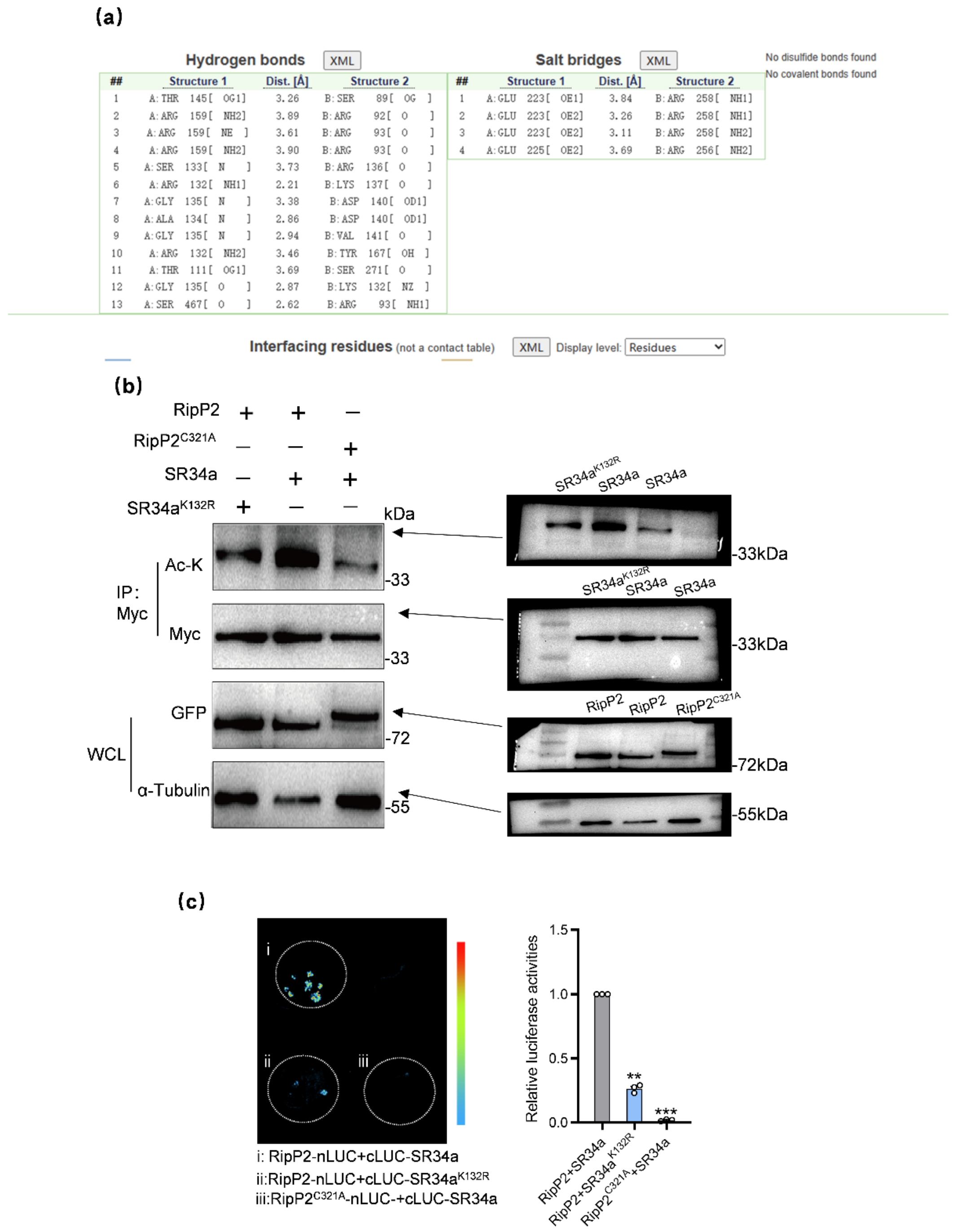

Hydrogen bonds XML

##	Structure 1	Dist. [Å]	Structure 2
1	A: THR 145[OG1]	3.26	B: SER 89[OG]
2	A: ARG 159[NH2]	3.89	B: ARG 92[O]
3	A: ARG 159[NE]	3.61	B: ARG 93[O]
4	A: ARG 159[NH2]	3.90	B: ARG 93[O]
5	A: SER 133[N]	3.73	B: ARG 136[O]
6	A: ARG 132[NH1]	2.21	B: LYS 137[O]
7	A: GLY 135[N]	3.38	B: ASP 140[OD1]
8	A: ALA 134[N]	2.86	B: ASP 140[OD1]
9	A: GLY 135[N]	2.94	B: VAL 141[O]
10	A: ARG 132[NH2]	3.46	B: TYR 167[OH]
11	A: THR 111[OG1]	3.69	B: SER 271[O]
12	A: GLY 135[O]	2.87	B: LYS 132[NZ]
13	A: SER 467[O]	2.62	B: ARG 93[NH1]

Salt bridges XML

##	Structure 1	Dist. [Å]	Structure 2
1	A: GLU 223[OE1]	3.84	B: ARG 258[NH1]
2	A: GLU 223[OE2]	3.26	B: ARG 258[NH1]
3	A: GLU 223[OE2]	3.11	B: ARG 258[NH2]
4	A: GLU 225[OE2]	3.69	B: ARG 256[NH2]

No disulfide bonds found
No covalent bonds found

Figure S5. Structural and functional analysis of RipP2-SR34a interaction and acetylation.
(a) Molecular docking predicted interaction sites between RipP2 (residues 111–159) and SR34a (residues 89–141), supported by a confidence score of 0.9604.
(b) In vivo acetylation assay in *Nicotiana benthamiana*. RipP2 acetylates SR34a at K132, confirmed by reduced acetylation signals with RipP2C321A (inactive mutant) or SR34aK132R (mutation at K132). Western blot verified consistent protein expression across all samples.
(c) LCI assay showed that SR34aK132R weakens the interaction with RipP2 and RipP2C321A weakens the interaction with SR34a.
